# Recreational Drug Overdose—Clinical Value of Toxicological Analysis

**DOI:** 10.3390/toxics12090662

**Published:** 2024-09-10

**Authors:** Tobias Zellner, Florian Eyer, Christian Rabe, Stefanie Geith, Bettina Haberl, Sabrina Schmoll

**Affiliations:** Division of Clinical Toxicology, Poison Centre Munich, School of Medicine and Health, Technical University of Munich, 81675 Munich, Germany; florian.eyer@tum.de (F.E.); christian.rabe@mri.tum.de (C.R.); stefanie.geith@mri.tum.de (S.G.); b.haberl@tum.de (B.H.); sabrina.schmoll@mri.tum.de (S.S.)

**Keywords:** toxicological analysis, clinical relevance, recreational drug overdose, opiate/opioid, benzodiazepine/Z-drug, NPS, Pregabalin

## Abstract

Background: Toxicological analysis of patients with acute recreational drug poisoning can improve our understanding of substance use patterns, clinical symptoms, and improve treatment. Patient history alone may be incomplete or misleading. The objective was to assess the differences in patient history and analytical results, to describe the clinical characteristics, implications and hospital management, and to describe the drug use pattern over time. Methods: A retrospective study including all patients admitted to our toxicology unit with recreational drug toxicity and analytical testing from October 2014 to December 2022. Results: 872 patients were included. Patient history revealed a median of one ingested substance class: opiates/opioids, benzodiazepines/Z-drugs, and Pregabalin were predominant. Urine analysis revealed a median of three ingested substance classes (*p* < 0.001). Benzodiazepines/Z-drugs, Pregabalin, and THC were severely underreported. Agitation and aggression, anxiety, hallucinations, and psychosis were frequent, associated with cocaine, cathinone/phenethylamine, and amphetamine/MDMA detection and required sedation. Coma was also frequent, associated with opiate/opioid, benzodiazepine/Z-drug, GBL/GHB, and Pregabalin detection and required intubation, and/or application of Naloxone and/or Flumazenil. Twelve patients arrived in cardiac arrest; all were positive for opiates/opioids. Four patients died: three with Benzodiazepines/Z-drugs, Pregabalin and opiates/opioids detected, one with cathinones/phenethylamines detected. While cathinones/phenethylamines and synthetic cannabinoid receptor agonists were mainly detected between 2014–2016, detection decreased significantly between 2017–2022 after NPS legislation passed. Pregabalin detection increased. Conclusions: Patient history is inaccurate, and patients frequently underreport ingested drugs. Opiates and opioids are still the main cause of morbidity and mortality. Pregabalin is increasingly abused. NPS legislation effectively decreased cathinone/phenethylamine and synthetic cannabinoid receptor agonist overdoses.

## 1. Introduction

Acute recreational drug toxicity is still a major public health issue and causes admissions to emergency departments (EDs) and intensive care units (ICUs). The European Monitoring Centre for Drugs and Drug Addiction (EMCDDA), now the European Union Drugs Agency, (EUDA) monitors the situation in Europe and publishes the European Drug Report on a yearly basis. The most recent European Drug Report 2024 emphasizes that the use of illicit drugs is now seen almost everywhere in our society [1]. The European Drug Emergencies Network (Euro-DEN and Euro-DEN plus) was established to collect data from 36 sentinel hospitals in 24 countries in the European Union, Switzerland, Georgia, and the United Kingdom [2,3]. The drugs recorded are based on the patient’s self-report and/or the clinical interpretation of the drugs by the clinicians caring for the patient. Therefore, the differences in patient history and toxicological investigations remain unclear.

During the last 20 years, new (novel) psychoactive substances (NPSs) like cathinones, phenethylamines, and synthetic cannabinoid receptor agonists (SCRAs) have gained popularity. This has posed an enormous challenge for clinicians, analytical scientists, law enforcement, and legislative authorities [4]. The EMCDDA is currently monitoring over 950 new psychoactive substances [1]. Data for forensic laboratories is collected and shared in accumulated mass spectra databases; however, the results are usually either not available to clinicians at all or not available in time for decision-making in treatment [5].

While the clinical characteristics of recreational drug toxicity have been described for, e.g., NPS [6], 3,4-methylenedioxy-methamphetamine (MDMA) [7], benzodiazepines/Z-drugs [8], gamma-butyrolactone (GBL)/gamma-hydroxybutyrate (GHB) [9], tetrahydrocannabinol (THC) [9], and the co-use of alcohol with CNS-depressants [10], all those studies rely on the patient’s self-report and/or the clinical interpretation of the drugs by the clinicians managing the patient.

Toxicological analysis is usually performed at a specialized laboratory and testing is often expensive. Emergency drug screenings are an alternative; however, the purpose of emergency drug screenings in EDs has also been questioned. Therefore, systematic toxicological analysis of patients with recreational drug toxicity is rarely performed [11]. Many clinics nowadays waive detailed toxicological testing or even emergency drug screenings. Toxicological testing therefore mainly serves an academic purpose. In times of healthcare economization, even this academic purpose has been scrutinized. Only through toxicological analysis, further knowledge about the clinical characteristics of drug use and drug use patterns over time can be evaluated properly—improving patient treatment.

Therefore, the aim of our study was:

1. To assess the differences in drug use between the patient history and analytical results;
2. To describe the clinical characteristics, implications, and hospital management of drug-intoxicated patients based on their analytical results;
3. To describe the drug use pattern over time.

## 2. Materials and Methods

We performed a retrospective, observational, monocentric study on patient data collected by our center for the Euro-DEN Plus project from October 2014 to December 2022. The study was conducted at the Toxicology Department of the Technical University of Munich, comprising a general ward, an intermediate care unit, and an intensive care unit (ICU). The study was conducted in accordance with the Declaration of Helsinki and approved by the Ethics Committee of the Technical University of Munich (protocol code 5916/13, 20 August 2013).

**Figure 1:**
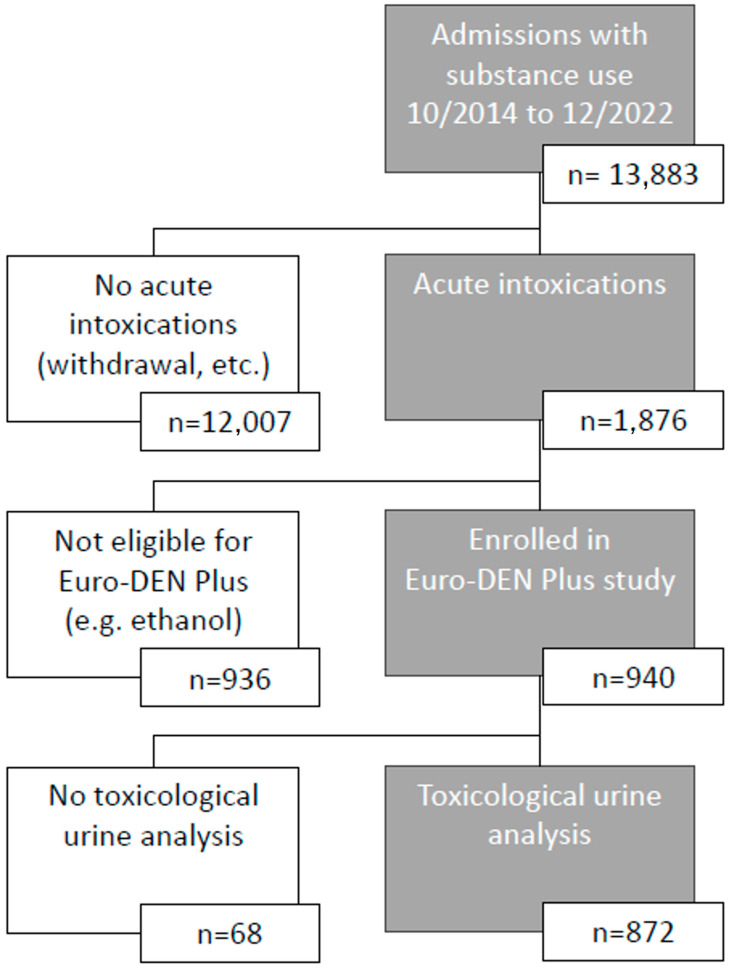
Patient enrollment.

All patients (including minors but at least 14 years old) with acute recreational drug toxicity or misuse of over-the-counter medicine for recreational purposes were included. Inclusion and exclusion followed the Euro-DEN methodology [2,3], enrollment data can be seen in Figure 1. Patients were either admitted directly, via Emergency Medical Services (EMSs), or via the ED. Urine samples for toxicological analysis were obtained as soon as possible, usually at the time of admission. Patients with lone ethanol intoxications, presentations not related to acute drug toxicity (e.g., infection, trauma, or drug withdrawal) or presentations related to self-harm were excluded. Patients without toxicological urine analysis were also excluded. Patients with multiple visits to our department within the study period were included as multiple cases. Data on patient demographics, clinical features, treatment, and outcome were collected by trained medical staff.

Ingested drugs were recorded according to the patient’s self-report on admission, bystander or relative reports (e.g., if the patient was in a coma), EMS statements, or clinical interpretation of the drugs by the clinicians managing the patient. To reflect reality in the ED, data were extracted from the admission report of the first treating physician. Later corrections were not considered, e.g., after the patient awoke from a coma. Ingested drugs were classified in substance classes (“history”): all opiates/opioids, Buprenorphine, Methadone, opiates, opioids (Fentanyl, Oxycodon, Tilidine, Tramadol), amphetamines/MDMA, benzodiazepines/Z-drugs (Zolpidem, Zopiclone), cathinones/phenethylamines, cocaine, GBL/GHB, Ketamine, Lysergic acid diethylamide (LSD), Pregabalin, SCRA, and THC. Analytical findings were recorded accordingly (“analytics”).

Urine analysis was performed for all patients with an immunoassay using an AU480 chemistry analyzer (Beckman Coulter, Brea, CA, USA) [12]. The test battery includes amphetamines/MDMA, Benzoylecgonine = cocaine, Buprenorphine, barbiturates, benzodiazepines, delta-9-THC, ethanol, Ethyl glucuronide, 2-Ethylidene-1,5-dimethyl-3,3-diphenylpyrrolidine = Methadone metabolite, Fentanyl, GHB, opiates, Pregabalin, Spice-1, Spice-2, and Spice-3. Dip tests were performed for selected substances (e.g., LSD, Ketamine, or Tilidine). From 2014 to mid-2020, online solid-phase extraction (SPE) high-performance liquid chromatography (HPLC)-diode array detector (DAD) TOX.I.S. II (Shimadzu, Kyoto, Japan) analysis was performed to detect substances not included in the immunoassay or to differentiate detailed substances. Using SPE-HPLC-DAD, it was possible to detect Oxycodon, Tilidine, Tramadol, Zolpidem, Zopiclone, certain cathinones/phenethylamines, and Ketamine, among other substances. From mid-2020 to the present, liquid chromatography mass spectrometry (LC-MS) using a ThermoFisher Ultimate 3000 HPLC with LTQ-XL Iontrap (ThermoFisher, Waltham, MA, USA) [13] replaced the TOX.I.S II and was supplemented with gas chromatography–mass spectrometry (GC MS) using an Agilent GC 8860 MSD 5977B (Agilent, Santa Clara, CA, USA) [14] in mid-2022 to perform general unknown screenings.

Data analysis was performed on demographic data (i.e., age and sex), management, outcome, and clinical characteristics. A Glasgow Coma Scale score of ≤8 was considered “coma”. Treatment included specific interventions, such as intubation, sedation, and antidote therapy or routine medical interventions (“any treatment”). Symptoms and treatment were correlated with substance classes detected in urine analysis. The outcome included length of hospital stay, discharge modality, or death.

Data were collected in Microsoft Excel (Microsoft, Redmond, WA, USA) and analyzed using IBM SPSS Version 29 (IBM, Armonk, New York, NY, USA). Qualitative variables were summarized using absolute numbers and percentages. Quantitative variables are displayed as the median plus interquartile range (IQR). Yearly statistics are displayed as average and minimum/maximum. Statistical differences were tested using the chi-square test for qualitative or the Wilcoxon–Mann–Whitney-U-test for quantitative variables. Positive and negative predictive values are displayed with their 95% confidence interval. Results were considered statistically significant if the *p* value was <0.05. Due to the exploratory nature of our study, adjustment for multiple testing was waived.

## 3. Results

Between October 2014 and December 2022, 872 patients were included in our study, 647 (74.2%) men and 225 (25.8% women). Their median age was 33 years (IQR 26–40). In total, 834 (95.6%) patients arrived via EMS. Enrollment data can be seen in Figure 1.

### 3.1. Patient History vs. Analytics

According to patient history, they had ingested a median of one (IQR 1–2) substance class excluding ethanol. Urine analysis revealed a median of three (IQR 2–4) ingested substance classes (*p* < 0.001). A total of 22.1% of patients tested positive for one substance class. In 2.2% of patients, no substance could be identified in the toxicological analysis. In two patients, eight substance classes could be detected. Ethanol was co-ingested in 361 (41.4%) cases.

Sex had no significant impact on drug detection, severity of intoxication, severe outcomes, or treatments like sedation or the application of Naloxon or Flumazenil. Men had a significantly higher need for intubation than women (8.7% vs. 4.2%, *p* = 0.048). There was no significant difference in discharge between men and women.

Amphetamines/MDMA, Buprenorphine, Methadone, opiates, opioids, benzodiazepines/Z-drugs, cocaine, GBL/GHB, Pregabalin, and THC were considerably underreported. The greatest difference between patient-reported and analytically confirmed drug classes was observed in benzodiazepines/Z-drugs, Pregabalin, and THC. NPS-like cathinones/phenethylamines and SCRA were slightly overreported.

The positive and negative predictive value (PPV and NPV) of patients reporting an ingestion of Buprenorphine, Methadone, opiates, Cocaine, and GBL/GHB was high. Patients reporting Pregabalin and THC ingestion had a high PPV. A missing report of Amphetamine/MDMA, cathinone/phenethylamine, Ketamine, LSD, opioid, and SCRA ingestion had a high NPV.

The differences between patient history and urine analysis are displayed in Table 1.

### 3.2. Symptoms

Agitation and aggression were the main symptoms in 330 (37.8%) cases. This was associated with the ingestion of amphetamines/MDMA (*p* < 0.001), cathinones/phenethylamines (*p* < 0.001), and cocaine (*p* = 0.010). In total, 176 (20.2%) patients reported anxiety, associated with the ingestion of amphetamines/MDMA (*p* < 0.001), cocaine (*p* < 0.001), and LSD (*p* < 0.001). Hallucinations were present in 156 (17.9%) patients, associated with amphetamine/MDMA (*p* < 0.001) and cathinone/phenethylamine (*p* < 0.001) toxicity. Psychosis was present in 79 (9.1%) patients, associated with the ingestion of cocaine (*p* = 0.032). All those symptoms were significantly less pronounced in patients testing positive for any opiate/opioid, benzodiazepines/Z-drugs, and Pregabalin. Agitation, aggression, and psychosis were also present in patients who tested positive with drugs not causing these symptoms (e.g., opiates and opioids). This can be due to polydrug use or the co-use of ethanol.

A total of 222 (25.5%) patients were comatose upon admission. This correlated significantly with the ingestion of benzodiazepines/Z-drugs (*p* = 0.001), GBL/GHB (*p* = 0.005), Pregabalin (*p* = 0.002), and opiates (*p* < 0.001). An additional 27 patients lost consciousness after admission, resulting in 249 (28.6%) patients with coma at any time during their hospitalization. Coma during treatment correlated with the same substance classes.

Cathinones/phenethylamine were also significantly associated with hyperthermia (*p* = 0.003) and showed a high rate of agitation and aggression in 67.1% of cathinone/phenethylamine-positive patients (*p* < 0.001). Hallucinations were also a prominent feature of cathinone/phenethylamine toxicity (38.6%, *p* < 0.001).

SCRA only showed a significantly increased rate of vomiting (*p* < 0.001) and seizures (*p* = 0.048). Agitation and aggression were also a prominent feature with 49.1% of SCRA-positive patients.

Twelve patients (1.4%) arrived in cardiac arrest and had to be resuscitated.

Patient symptoms and symptoms within selected substance classes of the whole cohort are shown in Table 2.

### 3.3. Treatment

EMS had already intubated 37 (4.2%) patients pre-clinically or applied Naloxone in 51 (5.8%) and/or Flumazenil in 19 (2.2%) cases. For 123 (14.1%) patients, EMS had started sedation.

In hospital, medical treatment was required for 846 (97.0%) patients (including standard diagnostics like physical exams, lab tests, and EKG and standard treatment like fluid replacement and vigilance and respiratory monitoring). An additional 24 patients (2.8%) were intubated, 45 (5.2%) received Naloxone, and 29 (3.3%) Flumazenil. Sedation was necessary in 289 (33.1%) patients.

Intubation in total was required for 61 (7.0%) patients and was significantly associated with the ingestion of GBL/GHB (*p* < 0.001). Naloxone was applied 89 (10.2%) times and was mainly given to opiate/opioid-positive patients (*p* < 0.001). Eleven patients received Naloxone without a positive urine test for opiates/opioids. Patients positive for benzodiazepine/Z-drugs and Pregabalin also received Naloxone significantly more frequently (*p* = 0.011 and *p* < 0.001). Flumazenil was applied 47 (5.4%) times. It was significantly more frequently applied to patients positive for benzodiazepine/Z-drugs, Pregabalin, and opiates/opioids (*p* < 0.001, *p* < 0.001, and *p* = 0.002). Since analytical results were not present at the time of antidote application, patients without, e.g., opiate/opioid toxicity might have received Naloxone.

Sedation was the main treatment modality and necessary in 342 (60.7%) patients. This was mainly due to amphetamine/MDMA, cathinone/phenethylamine, and cocaine toxicity (*p* < 0.001, *p* = 0.019, and *p* < 0.001). Midazolam, Propofol, Clonidine, Dexmeditomidine, and Esketamine were the main sedatives and were combined with Haloperidol or Olanzapine if patients presented with psychotic symptoms.

Treatment for all patients and for patients within selected substance classes is displayed in Table 3.

### 3.4. Outcome

Overall, the median duration of a patient’s treatment was 19 h (IQR 11–61). Most patients were discharged against medical advice (599 patients), 170 (19.5%) patients were discharged regularly, 58 (6.7%) patients were referred to a psychiatric hospital, and 41 (4.7%) to another medical facility (e.g., rehabilitation).

All patients with cardiac arrest on admission tested positive for opiates or opioids (*p* = 0.003) with opiates present in 83.3% of cases (*p* < 0.001). Pregabalin was also significantly overrepresented in patients with cardiac arrest (*p* = 0.032).

Four (0.5%) patients died. In three patients, benzodiazepines/Z-drugs and opiates (*p* = 0.041) were detected, another patient died due to amphetamines/MDMA and cathinone (Methylon) toxicity.

Analytical results for patients arriving with cardiac arrest and those who died is shown in Table 4.

### 3.5. Analytical Trends over Time

While most substance classes remained stable over the years, some substance classes showed an increase or decrease. Benzodiazepines/Z-drugs were present on average in 54.8% of samples (43.0–65.2%) with a peak in 2020 and an overall increase. The same applies for Pregabalin which was found in average in 46.2% of patients (30.6–52.3%) with a peak in 2017.

Amphetamines/MDMA were found in 21.4% (16.7–30.0%) cases with a peak in 2021. Cocaine was present in 17.5% (9.6–22.9%) of samples with a peak in 2021. Both cocaine and amphetamines/MDMA varied without a clear trend over time. GBL/GHB varied over the years with an average of 3.7% (1.4–6.5%) and a peak in 2017. Opiates/opioids were the most frequently detected substances and were on average present in 58% of patient samples (51.4–61.7%) with a peak in 2020. THC remained stable with an average of 38% (31.7–45.1%) of positive tests.

NPSs like cathinones/phenethylamines were found on average in 5.5% of cases with a maximum of 14.6% in 2015 and were not detected in 2020. SCRA was found on average in 5.2% of cases with a maximum of 13.2% in 2016 and was not detected in 2022. While cathinones/phenethylamines and SCRA were detected in 17.9% and 9.9% of patients between 2014–2016, they were only detected in 2.8% and 4.0% between 2017 and 2022 after NPS legislation passed in Germany (*p* < 0.001).

**Figure 2:**
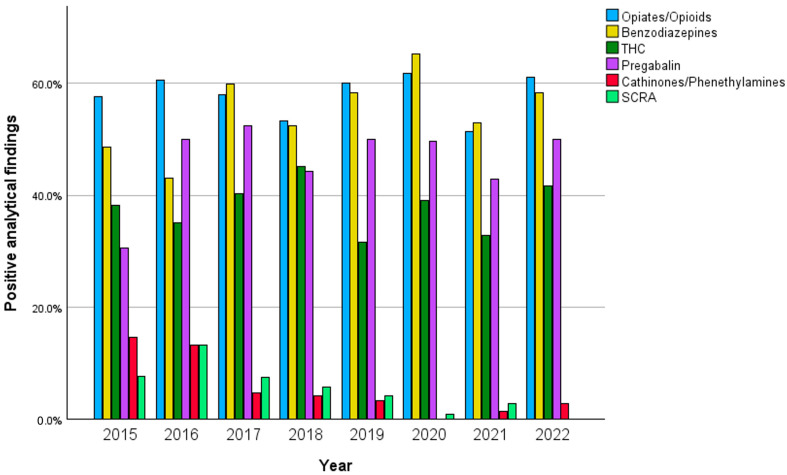
Timeline of selected substance classes per year in percentages.

The trend of selected substance classes over time can be seen in Figure 2.

## 4. Discussion

The main findings of our study were that patient history inaccurately describes drug ingestions: benzodiazepines/Z-drugs, Pregabalin, and THC were especially underreported. Furthermore, benzodiazepines/Z-drugs and Pregabalin are increasingly abused. NPS legislation in Germany effectively decreased cathinone/phenethylamine and SCRA overdoses. Agitation and aggression, anxiety, hallucinations, and psychosis as well as coma were frequent symptoms. Sedation, antidote therapy with Naloxone and/or Flumazenil, or intubation were often required. Opiates and opioids were the main cause of morbidity and mortality.

Our findings show that there is a significant difference between patient history and analytical findings. Patients reported a median of one (IQR 1–2) ingested substance classes, analytical testing revealed a median of three (IQR 2–4) substance classes. In total, 22.1% of patients tested positive for one substance class. In 2.2% of patients, no substance could be identified through toxicological analysis. In two patients, eight substance classes could be detected. There may be numerous reasons for this finding. Incomplete or inaccurate patient histories might have played a role and are a common problem in patients with drug ingestion [15,16]. Patients might only report the main substance responsible for their overdose and leave out co-ingested drugs like be benzodiazepines/Z-drugs, Pregabalin, and THC which they might deem irrelevant and therefore have a low NPV. Patients might also have been unaware of consuming additional drugs that were detected in their system. Most established drugs like amphetamines/MDMA, benzodiazepines/Z-drugs, Buprenorphine, cocaine, Methadone, opiates, Pregabalin, and THC have a high PPV. For these substance classes, we can assume that patients knew what they ingested, and this was subsequently confirmed by analytical testing. Newer substance classes like cathinones/phenethylamines and SCRA as well as Ketamine and LSD had a low PPV. For these substances, patients themselves were often unaware of what exactly they had ingested. In particular, new cathinones/phenethylamines and SCRA posed a great challenge for analytical testing because of their sometimes unknown structure and their absence in available mass spectral libraries and databases. Therefore, there is a delay between the appearance of these substances on the market and possible analytical testing [17]. NPSs are constantly evolving as alternatives to classic drugs—containing more effective substances with higher receptor affinity—leading to health care risks and posing a challenge for medical service analytical testing and legislation [5,18]. These findings are in line with Liakoni et al., who described a high analytical confirmation for heroin and cocaine and an advantage for mass spectrometry in detecting NPSs [19]. Bharat et al. describes an even higher association between self-reporting and biological testing; however, this meta-analysis was not performed in a clinical setting. It has also been shown that sensitivity estimates differ between research studies with no consequences and studies conducted in criminal justice settings [20]. Our study focused on patient reports submitted at the time of admission which is what clinicians are confronted with. Reports might have been more accurate if patients were interviewed after their acute intoxication had passed. Overall, focusing on the patients’ history alone may only represent a relatively small spectrum of reality. This is the methodology used by the Euro-DEN-Plus study group [21]. To make the study results even more robust, analytical confirmation would be desirable and could improve the validity of the results. Using our study data and the PPV and NPV of certain substance classes, we can, e.g., assume that studies like the one by Galicia et al., describing the clinical relevance of ethanol coingestion in patients with GBL/GHB intoxication, have a high validity (GBL/GHB PPV 94.1%, NPV 98.1%) [9]. This study might help with the interpretation of further Euro-DEN-Plus publications.

Urine analytics showed a clear predominance of sedatives with opiates/opioids in 57.2% of cases, followed by benzodiazepines (55.6%), Pregabalin (44.4%), THC (37.7%), and GBL/GHB (3.7%), leading to typical symptoms like coma and CNS depression. While ethanol was only co-ingested in 41.4% of cases, it was shown that ethanol co-ingestion significantly increased the risk of severe outcomes in CNS-depressing substances [10]. Sympathomimetic substances like amphetamines/MDMA (21.3%), cathinones/phenethylamines (8.0%), and cocaine (16.7%) were associated with agitation and aggression. This was also true for and SCRA (6.1%). This underlines the findings of several studies like Crulli et al., Noseda et al., Romanek et al., and Schmoll et al., showing violent thoughts and acts in one-third of users of synthetic cathinones as well as prolonged confusion as the most common complications [6,7,22,23]. Vallersnes et al. showed that psychosis was significantly associated with amphetamine, methylenedioxypyrovalerone, SCRA, tryptamine, and LSD toxicity [24]. SCRA consumption led to significantly increased rates of vomiting, seizures, and arrhythmias, also described by Waters et al. showing significantly higher rates of drowsiness, coma, agitation, seizures, and bradycardia for intoxication with lone synthetic cannabinoid receptor agonist exposures compared with cannabis use [25]. Hallucinations and psychosis associated with the use of amphetamines/MDMA and cathinones/phenethylamines as well as psychosis after cocaine ingestion were significantly reduced in polyvalent drug misuse. This was also especially true for patients who tested positive for benzodiazepines/Z-drugs, Pregabalin, and opiate/opioids—assuming an antagonistic effect of these drugs and corresponding with the findings of Heier et al. [10]. Overall, our study confirms the findings of previous studies where patient records and not urine analysis were used as a reference.

Only 22.1% of patients tested positive for one substance—predominantly amphetamines/MDMA (35 patients) and THC (43 patients)—confirming that polyvalent drug abuse predominates in Europe [1]. Polydrug use is a main risk factor for ICU requirement in recreational drug toxicity as shown by Noseda et al. [26]. In addition to trends like polyvalent substance use, the European Drug Report 2014 stresses the risks of highly potent substances, e.g., nitazene, and an overall rapidly growing market. According to the reported European drug seizures, the amount of synthetic cathinones and cannabinoids is still high, but synthetic opioids and benzodiazepines are increasing [1]. Nevertheless, cathinone/phenethylamine and SCRA decreased significantly in our center after Germany passed NPS legislation in November 2016 [27]. This law added NPS-like cathinones, phenethylamines, and SCRA, among others, to the list of controlled substances and seemed to have had an effect in our study population.

In suspected opiate/opioid overdose, application of Naloxone—the antagonist of opiates and opioids for all opioid receptors—can be considered. In benzodiazepine/z-substance toxicity, Flumazenil—the antagonist of benzodiazepines at the GABA_A_-receptor—can be applied. Naloxone was applied more frequently (10.2%) compared to Flumazenil (5.4%). This is in line with current recommendations, reinforcing that Flumazenil should not be applied routinely due to severe adverse effects like triggering withdrawal syndrome or seizures [28]. The clinical identification of opiate/opioid or benzodiazepine/z-substance toxicity and the subsequent application of Naloxone or Flumazenil was correct in 87.6% and 83% of cases. Naloxone and Flumazenil together were applied in 23 cases for unknown coma. EMSs and clinicians apparently had a good understanding of drug toxicity or used Naloxone as diagnostic tool. Because of the novel highly potent synthetic opioids and benzodiazepines, it is unknown if higher doses of Naloxone or Flumazenil are needed to show a clinical effect [29]. Unfortunately, Naloxon dose was not investigated in our study. However, applying doses higher than 0.4 mg is uncommon in our department. In cases where antagonization with significantly more than 0.4 mg naloxone is required, intubation, sedation, and ventilation may be carried out at the discretion of the treating physician.

While self-discharge rates vary between countries and hospitals, the average among Euro-DEN emergency rooms was reported at 1/8th [30]. Our high rate of 68.7% might be explained by the fact that all patients were offered to stay for detoxification. By refusing detoxification and requesting discharge after the acute intoxication had been treated, discharge was considered “against medical advice”.

Although our findings demonstrate the importance of analytical drug screening in recreational drug poisonings, these methods are rarely routinely available—mainly due to high costs (e.g., equipment, solvents, specialized personnel) and long analytical run length with delayed results often arriving after primary care has been completed or the patient has even already left the hospital due to short stays (median treatment time 19 h) [31] To avoid missing substances and to maximize the number of tested substances, several analytical methods should be combined. The hospital management of intoxicated patients should not be delayed because of analytical testing.

## 5. Limitations

The main limitation of the study is the monocentric, retrospective design. To confirm our findings, a multicenter study design would be desirable. Another limitation is the missing standardization in time from clinical presentation to sample preservation for analytics. Substances like GBL/GHB, known to have a short detection time in blood and urine from 6 to 10 h, may be underrepresented [31,32]. On the other hand, substances like benzodiazepines with long elimination half-lives could be overrepresented.

Another limitation is the methodology used for finding synthetic cannabinoids by using Spice-1, Spice-2, and Spice-3 testing via immunoassay—leading to a limited spectrum detected. This also plays an important role in the finding of novel synthetic drugs, especially cathinones and opioids. Since different analytical tools were used over time, this might have influenced the trends of drug use over time, especially concerning NPS and SCRA.

## 6. Conclusions

Patient history is often inaccurate, and patients frequently underreport ingested drugs, especially benzodiazepines/Z-drugs, Pregabalin, and THC. Opiates and opioids are still the main cause of morbidity and mortality. Pregabalin is increasingly abused. NPS legislation in Germany effectively decreased cathinone/phenethylamine and SCRA overdoses.

Without toxicological analysis, these findings would not be possible. Large data collections like the *European Drug Report* would be more inaccurate. Therefore, we encourage other clinics and academic institutions to perform analytical testing. We would encourage further research in faster and more reliable analysis (e.g., advanced point-of-care testing).

## Figures and Tables

**Table 1:** Patient history vs. analytics.

Substance Class		History	Analytics	PPV	NPV
All opiates and opioids	Yes	322 (36.9%)	499 (57.2%)	93.8% (90.6–95.9%)	64.2% (60.1–68.1%)
	No	550 (63.1%)	373 (42.8%)		
Buprenorphine	Yes	51 (5.8%)	157 (18.0%)	82.4% (69.7–90.4%)	86.0% (83.5–88.2%)
	No	821 (94.2%)	715 (82.0%)		
Methadone	Yes	109 (12.5%)	194 (22.2%)	88.1% (80.7–92.9%)	87.2% (84.6–89.4%)
	No	763 (87.5%)	678 (77.8%)		
Opiates	Yes	162 (18.6%)	252 (28.9%)	78.4% (71.4–84.0%)	82.4% (79.4–85.1%)
	No	710 (81.4%)	620 (71.1%)		
Opioids	Yes	35 (4.0%)	67 (7.7%)	51.4% (35.6–67.0%)	94.1% (92.4–95.6%)
	No	837 (96.0%)	805 (92.3%)		
Amphetamines and MDMA	Yes	116 (13.3%)	186 (21.3%)	73.3% (64.6–80.5%)	86.6% (84.0–88.9%)
	No	756 (86.7%)	686 (78.7%)		
Benzodiazepines and Z-drugs	Yes	181 (20.8%)	467 (53.6%)	96.1% (92.2–98.1%)	57.6% (53.4–60.7%)
	No	691 (79.2%)	405 (46.4%)		
Cathinones and phenethylamines	Yes	92 (10.6%)	70 (8.0%)	41.3% (31.8–51.5%)	95.9% (94.3–97.1%)
	No	780 (89.4%)	802 (92.0%)		
Cocaine	Yes	76 (8.7%)	146 (16.7%)	78.9% (68.5–86.6%)	89.2% (86.9–91.2%)
	No	796 (91.3%)	726 (83.3%)		
GBL/GHB	Yes	17 (1.9%)	32 (3.7%)	94.1% (73.0–99.0%)	98.1% (97.0–98.9%)
	No	855 (98.1%)	840 (93.3%)		
Ketamine	Yes	6 (0.7%)	4 (0.5%)	50% (18.8–81.2%)	99.9% (99.3–100%)
	No	866 (99.3%)	868 (99.5%)		
LSD	Yes	17 (1.9%)	9 (1.0%)	47.1% (26.2–69.0%)	99.9% (99.4–100%)
	No	855 (98.1%)	863 (99.0%)		
Pregabalin	Yes	131 (15%)	387 (44.4%)	90.1% (83.8–94.1%)	63.7% (60.2–67.1%)
	No	741 (85.0%)	485 (55.6%)		
SCRA	Yes	71 (8.1%)	53 (6.1%)	50.7% (39.3–62.0%)	97.9% (96.6–98.7%)
	No	801 (91.9%)	819 (93.9%)		
THC	Yes	86 (9.9%)	329 (37.7%)	80.2% (70.6–87.3%)	66.9% (63.6–70.1%)
	No	786 (90.1%)	543 (62.3%)		

PPV = positive predictive value, NPV = negative predictive value.

**Table 2:** Symptoms in all patients and in selected substance classes.

Symptoms		All *n* = 872	All Opiates and Opioids *n* = 499	*p*	BZD and Z-Drugs *n* = 467	*p*	Cathinones and PA *n* = 70	*p*	SCRA *n* = 53	*p*
Coma on admission	Yes	222 (25.5%)	150 (30.1%)	<0.001 *	140 (30.0%)	0.001 *	18 (25.7%)	0.959	9 (17.0%)	0.144
	No	650 (74.5%)	349 (69.9%)		327 (70.0%)		52 (74.3%)		44 (83.0%)	
Vomiting	Yes	40 (4.6%)	18 (3.6%)	0.110	15 (3.2%)	0.037 *	5 (7.1%)	0.287	8 (15.1%)	<0.001 *
	No	832 (95.4%)	481 (96.4%)		452 (96.8%)		65 (92.9%)		45 (84.9%)	
Hyperthermia	Yes	32 (3.7%)	22 (4.4%)	0.179	19 (4.1%)	0.501	7 (10.0%)	0.003 *	3 (5.7%)	0.426
	No	840 (96.3%)	477 (95.6%)		448 (95.9%)		63 (90.0%)		50 (94.3%)	
Headache	Yes	17 (1.9%)	5 (1.0%)	0.019 *	6 (1.3%)	0.127	1 (1.4%)	0.742	2 (3.8%)	0.322
	No	855 (98.1%)	494 (99.0%)		461 (98.7%)		69 (98.6%)		51 (96.2%)	
Anxiety	Yes	176 (20.2%)	51 (10.2%)	<0.001 *	51 (10.9%)	<0.001 *	20 (28.6%)	0.068	12 (22.6%)	0.645
	No	696 (79.8%)	448 (89.8%)		416 (89.1%)		50 (71.4%)		41 (77.4%)	
Hallucinations	Yes	156 (17.9%)	50 (10.0%)	<0.001 *	50 (10.7%)	<0.001 *	27 (38.6%)	<0.001 *	14 (26.4%)	0.095
	No	716 (82.1%)	449 (90.0%)		417 (89.3%)		43 (61.4%)		39 (73.6%)	
Agitation and aggression	Yes	330 (37.8%)	119 (23.8%)	<0.001 *	125 (26.8%)	<0.001 *	47 (67.1%)	<0.001 *	26 (49.1%)	0.082
	No	542 (62.2%)	380 (76.2%)		342 (73.2%)		23 (32.9%)		27 (50.9%)	
Psychosis	Yes	79 (9.1%)	26 (5.2%)	<0.001 *	25 (5.4%)	<0.001 *	10 (14.3%)	0.112	6 (11.3%)	0.554
	No	793 (90.9%)	473 (94.8%)		442 (94.6%)		60 (85.7%)		47 (88.7%)	
Seizures	Yes	58 (9.1%)	25 (5.0%)	0.024 *	20 (4.3%)	0.003 *	6 (8.6%)	0.501	7 (13.2%)	0.048 *
	No	793 (90.9%)	474 (95.0%)		447 (95.7%)		64 (91.4%)		46 (86.8%)	
Palpitations	Yes	36 (4.1%)	8 (1.6%)	<0.001 *	6 (1.3%)	<0.001 *	4 (5.7%)	0.487	1 (1.9%)	0.397
	No	836 (95.9%)	491 (98.4%)		461 (98.7%)		66 (94.3%)		52 (98.1%)	
Chest pain	Yes	24 (2.8%)	11 (2.2%)	0.253	7 (1.5%)	0.015 *	3 (4.3%)	0.414	1 (1.9%)	0.691
	No	848 (97.2%)	488 (97.8%)		460 (98.5%)		67 (95.7%)		52 (98.1%)	
Hypertension	Yes	35 (4.0%)	13 (2.6%)	0.014 *	9 (1.9%)	<0.001 *	1 (1.4%)	0.251	2 (3.8%)	0.927
	No	837 (96.0%)	486 (97.4%)		458 (98.1%)		69 (98.6%)		51 (96.2%)	
Hypotension	Yes	50 (5.7%)	34 (6.8%)	0.113	31 (6.6%)	0.217	4 (5.7%)	0.994	6 (11.3%)	0.071
	No	822 (94.3%)	465 (93.2%)		436 (93.4%)		66 (94.3%)		47 (88.7%)	
Arrhythmias	Yes	8 (0.9%)	5 (1.0%)	0.762	3 (0.6%)	0.360	0 (0%)	0.401	3 (5.7%)	<0.001 *
	No	864 (99.1%)	494 (99.0%)		464 (99.4%)		70 (100%)		50 (94.3%)	
Coma during treatment	Yes	249 (28.6%)	166 (33.3%)	<0.001 *	155 (33.2%)	0.001 *	25 (36.2%)	0.145	11 (21.2%)	0.219
	No	621 (71.2%)	332 (66.7%)		312 (66.8%)		44 (63.8%)		41 (78.8%)	

BZD = benzodiazepines, PA = Phenethylamines. Bold * = *p* < 0.05.

**Table 3:** Treatment for all patients and in selected substance classes.

Treatment		All *n* = 872	All Opiates and Opioids *n* = 499	*p*	BZD and Z-Drugs *n* = 467	*p*	Cathinones and PA *n* = 70	*p*	SCRA *n* = 53	*p*
Treatment	Yes	846 (97.0%)	492 (98.6%)	0.002 *	461 (98.7%)	0.002 *	69 (98.6%)	0.426	46 (86.8%)	<0.001 *
	No	26 (3.0%)	7 (1.4%)		6 (1.3%)		1 (1.4%)		7 (13.2%)	
Intubation	Pre-hospital	37 (4.2%)	25 (5.0%)	0.057	21 (4.5%)	0.723	3 (4.3%)	0.304	1 (1.9%)	0.694
	Hospital	24 (2.8%)	17 (3.4%)		13 (2.8%)		4 (5.7%)		2 (3.8%)	
	No	811 (93.0%)	457 (91.6%)		433 (92.7%)		63 (90.0%)		50 (94.3%)	
Naloxone	Pre-hospital	44 (5.0%)	39 (7.8%)	<0.001 *	26 (5.6%)	0.011 *	2 (2.9%)	0.638	0 (0%)	**0.011**
	Hospital	38 (4.4%)	32 (6.4%)		29 (6.2%)		4 (5.7%)		0 (0%)	
	Both	7 (0.8%)	7 (1.4%)		4 (0.9%)		0 (0%)		0 (0%)	
	No	783 (89.8%)	421 (84.4%)		408 (87.4%)		64 (91.4%)		53 (100%)	
Flumazenil	Pre-hospital	18 (2.1%)	13 (2.6%)	0.002 *	14 (3.0%)	<0.001 *	0 (0%)	0.878	0 (0%)	0.076
	Hospital	28 (3.2%)	23 (4.6%)		24 (5.1%)		4 (5.8%)		0 (0%)	
	Both	1 (0.1%)	1 (0.2%)		1 (0.2%)		0 (0%)		0 (0%)	
	No	824 (94.5%)	462 (92.6%)		428 (91.6%)		65 (94.2%)		52 (100%)	
Sedation	Pre-hospital	53 (6.1%)	22 (4.4%)	<0.001 *	11 (2.4%)	<0.001 *	5 (7.1%)	<0.001 *	10 (18.9%)	<0.001 *
	Hospital	219 (25.1%)	90 (18.1%)		95 (20.3%)		27 (38.6%)		9 (17.0%)	
	Both	70 (8.0%)	30 (6.0%)		27 (5.8%)		11 (15.7%)		3 (5.7%)	
	No	529 (60.7%)	356 (71.5%)		334 (71.5%)		27 (38.6%)		31 (58.5%)	

BZD = benzodiazepines, PA = Phenethylamines. Bold * = *p* < 0.05

**Table 4:** Analytical results for patients with cardiac arrest on admission or death.

Substance Class		Cardiac Arrest *n* = 12	*p*	Death *n* = 4	*p*
All opiates and opioids	Yes	12 (100%)	0.003 *	3 (75.0%)	0.471
	No	0 (0%)		1 (25.0%)	
Buprenorphine	Yes	3 (25.0%)	0.525	1 (25.0%)	0.715
	No	9 (75.0%)		3 (75.0%)	
Methadone	Yes	2 (16.7%)	0.640	0 (0%)	0.284
	No	10 (83.3%)		4 (100%)	
Opiates	Yes	10 (83.3%)	<0.001 *	3 (75.0%)	0.041 *
	No	2 (16.7%)		1 (25.0%)	
Opioids	Yes	2 (16.7%)	0.239	0 (0%)	0.563
	No	10 (83.3%)		4 (100%)	
Amphetamines and MDMA	Yes	0 (0%)	0.069	1 (25.0%)	0.862
	No	12 (100%)		3 (75.0%)	
Benzodiazepines and Z-drugs	Yes	9 (75.0%)	0.134	3 (75.0%)	0.389
	No	3 (25.0%)		1 (25.0%)	
Cathinones and phenethylamines	Yes	0 (0%)	0.303	1 (25.0%)	0.211
	No	12 (100%)		3 (75.0%)	
Cocaine	Yes	2 (16.7%)	0.994	0 (0%)	0.369
	No	10 (83.3%)		4 (100%)	
Ethanol	Yes	5 (41.7%)	0.985	1 (25.0%)	0.505
	No	7 (58.3%)		3 (75.0%)	
GBL/GHB	Yes	0 (0%)	0.496	0 (0%)	0.696
	No	12 (100%)		4 (100%)	
Ketamine	Yes	0 (0%)	0.813	0 (0%)	0.892
	No	12 (100%)		4 (100%)	
LSD	Yes	0 (0%)	0.722	0 (0%)	0.838
	No	12 (100%)		4 (100%)	
Pregabalin	Yes	9 (75.0%)	0.032 *	2 (50.0%)	0.817
	No	3 (25.0%)		2 (50.0%)	
SCRA	Yes	0 (0%)	0.375	0 (0%)	0.610
	No	12 (100%)		4 (100%)	
THC	Yes	3 (25.0%)	0.360	0 (0%)	0.119
	No	9 (75.0%)		4 (100%)	

Bold * = *p* < 0.05.

## Data Availability

Data can be requested from the corresponding author.
